# Distal Triceps Tendon Repair in Strength Athletes Leads to Satisfactory Return to Sports: A Retrospective Analysis of 22 Cases

**DOI:** 10.3390/jcm13164913

**Published:** 2024-08-20

**Authors:** Michael Stephan Gruber, Martin Bischofreiter, Felix Rittenschober, Michael Schachermayr, Reinhold Ortmaier, Mathias Ritsch

**Affiliations:** 1 Department of Orthopedic Surgery, Ordensklinikum Linz Barmherzige Schwestern, Vinzenzgruppe Center of Orthopedic Excellence, Teaching Hospital of the Paracelsus Medical University, 5020 Salzburg, Austria; 2 Medical Faculty, Johannes Kepler University Linz, Altenbergerstraße 69, 4040 Linz, Austria; 3 Sportortho Rosenheim, 83022 Rosenheim, Germany

**Keywords:** distal triceps repair, triceps rupture, chronic tendon injury, triceps reconstruction, DTTR

## Abstract

**Background**: Distal triceps brachii tendon rupture (DTTR) is a relatively rare injury that is common in bodybuilding and high-intensity contact sports and can lead to significant functional impairment of the elbow joint. This study was conducted to evaluate clinical outcomes and the rate of return to sports among competitive bodybuilders and high-demand strength athletes after surgical repair of DTTR. **Methods**: This retrospective case series was performed in an institutional setting in tertiary health care. Return to sports of 22 competing or high-demand strength athletes (meaning three or more exercises per week) were analyzed pre- and postoperatively after surgical repair of DTTR using a hybrid technique of transosseous sutures and anchor fixation. Descriptive statistics were used to analyze demographic variables, and independent and paired *t*-tests were used to assess clinical outcomes. **Results**: The follow-up showed that from pre- to postoperatively, there was no deterioration in the number of sports disciplines (2.4 and 2.5 sporting activities per person, respectively; *p* = 0.540) or in the frequency of weekly training (4.1 and 4.1 times per person, respectively; *p* = 0.329). The postoperative visual analog scale for pain (from 6.0 to 1.6, *p* < 0.001), level of fitness (from 5.1 to 2.6, *p* = 0.002), and ability to train (from 5.2 to 1.3, *p* < 0.001) improved significantly. The time of return to sports was 1.5–3 months and 4–6 months after the surgery for ten patients each. The overall rate of return to sports was 95%, whereas 86% returned to the preinjury level of sporting activity. **Conclusions**: Repair of DTTR leads to high rates of return to sports in competitive athletes.

## 1. Introduction

Distal triceps brachii tendon rupture (DTTR) is a rare condition that affects mostly males between the ages of 30 and 60 years [1,2]. Although DTTR accounts for less than 1% of all upper limb tendon ruptures [3,4], the development and practice of high-intensity contact sports such as American football or service in the army may increase the risk of DTTR in those individuals [5]. DTTR can occur at different anatomical locations as a tear at the insertion, as an intratendinous rupture, or at the musculotendinous junction. The tear can involve the deep or the superficial layer alone, or it occurs as a full-thickness tear; however, it most commonly occurs at or around the tendon’s insertion onto the olecranon. Factors such as pre-existing chronic bursitis of the elbow and anabolic steroid misuse increase the risk of DTTR [6,7,8]. The mechanism of injury typically involves a high load on the extended elbow or a jerky concentric contraction of the triceps brachii, although cases have also been described after motor vehicle accidents [9].

There are several surgical techniques used for refixation of the distal triceps tendon, each with specific advantages and disadvantages. The most commonly used techniques are the anchor technique, in which anchors are inserted into the olecranon and used to fix the torn tendon, transosseous refixation, in which the tendon is fixed using sutures that are passed through drill holes in the olecranon, and hybrid methods combining the two techniques mentioned. While all methods show comparable clinical outcomes [10], anchors seem to result in lower re-rupture and reoperation rates [11,12]. All techniques mentioned can be performed as a single or double row; however, the latter leads to biomechanical advantages compared to the former [13,14].

Despite the available data on surgical techniques and the clinical and functional outcomes of DTTR [12], there remain significant gaps in the literature, particularly concerning return to sports and specific surgical outcomes. Most of the existing studies focus on the general clinical and functional recovery of patients, often neglecting detailed evaluations of return-to-sport metrics, which are crucial for high-demand athletes. There is a lack of standardized criteria for assessing successful return to sports, and many studies do not account for the varying demands of different sports on the repaired tendon [15,16].

Furthermore, the literature provides limited insights into the long-term outcomes of surgical repair in athletes, such as the durability of the repair under repeated stress and the potential for re-injury. The psychological aspect of recovery, including confidence in the repaired tendon and its impact on athletic performance, is also under-researched [17,18].

While some studies provide limited information on return to work and general physical activity post-surgery, comprehensive data on the return to competitive sports, especially in high-demand strength athletes, are scarce [19,20,21,22]. This study aims to address these gaps by evaluating the clinical outcomes and rate of return to sports among competitive bodybuilders and high-demand strength athletes following surgical repair of DTTR. It was designed to analyze the outcome of a certain technique (hybrid technique) in a certain population (competitive bodybuilders and high-demand strength athletes). The hypothesis is that surgical repair of DTTR fully restores sportive function in this population.

## 2. Materials and Methods

This study was performed according to the REporting of studies Conducted using Observational Routinely collected Data (RECORD) guidelines. All subjects gave their informed consent for inclusion before participating in the study.

Patients who underwent surgical repair of distal triceps tendon rupture in the period from 2010 to 2019 were retrospectively evaluated. Inclusion criteria were (1) surgical repair of DTTR, (2) competitive strength athletes or a minimum of three exercises per week, and (3) a minimum follow-up of 12 months after surgery. The follow-up examination was conducted using a questionnaire evaluating pre-operative and postoperative sports activities. Exclusion criteria were (1) previous surgery on the affected elbow, (2) further injuries to the elbow other than DTTR, and (3) missing consent to participate.

Altogether, 70 patients were treated with the surgery in question, and 44 were eligible to participate after applying inclusion and exclusion criteria. The level of performance was self-reported and categorized into professional, recreational, or none, and the same applied to the frequency of exercises per week. However, only 22 of the 44 patients could be followed up. This was mainly due to failure to return the questionnaire. No differences between the patients who did not participate and those who participated were detected. To determine age-dependent differences, the median age was calculated, and the patients were divided into subgroups accordingly (<45 years and ≥45 years).

### 2.1. Surgical Technique

The operations were performed under general anesthesia with the patient in the prone position and the affected arm abducted. The skin incision was approximately 6 cm long, starting at the distal third of the triceps and then going laterally around the olecranon until reaching 1.5 cm distal to the tip of the olecranon. Partial ruptures were completed or left in place, depending on the configuration. The injured tendon was carefully dissected, and any scar tissue or debris was removed to allow a better view of the tear. Debridement of the olecranon followed.

**Figure 1:**
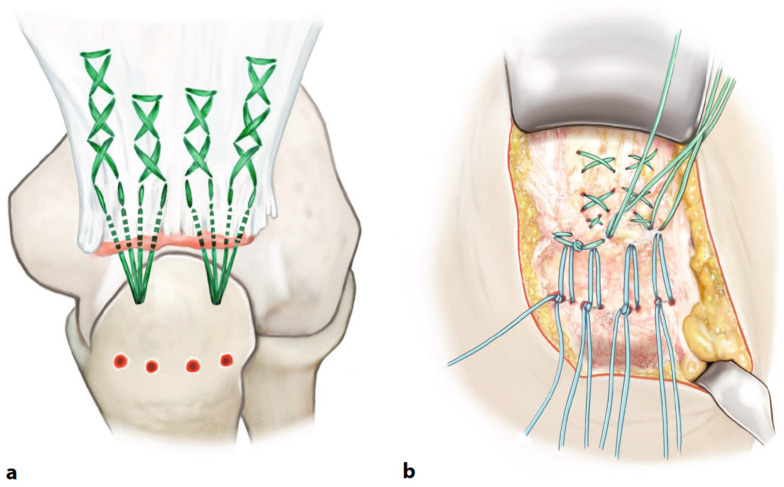
Titanium or all-suture anchors in place with whipstitches used to fix the deep and the superficial tendon layer ((**a**) green sutures). Predrilled holes for transosseous sutures (in blue) to brace the superficial leaflet as a suture bridge (**b**). Reprinted with permission of Mathias Ritsch [23].

After mobilization of the tendon stump, 2 × 2.9 mm all-suture or titanium anchors were placed in the olecranon after predrilling. The choice of which anchor was used was based on the quality of the bony anchorage; all-suture anchors were generally used (JuggerKnot^®^, Zimmer-Biomet, Warsaw, IN, USA), and titanium anchors were used for poor bone stock (Super Quickanker plus^®^, Mitek—DePuy Synthes, Raynham, MA, USA). In addition, two to four holes for transosseous sutures were drilled 12 mm distal to the tip of the olecranon, and transosseous sutures were placed. The anchor sutures were then used to fix the deep and superficial tendon leaflets to the olecranon, and the transosseous sutures were used to additionally brace the peripheral portions of the superficial tendon leaflet as a bridge system (Figure 1) [23].

After completion of the repair, the incision was closed with sutures, a sterile dressing was applied to the wound, and a splint was applied in a 10° extension position while the patient was still in the operating room.

### 2.2. Postoperative Rehab

Postoperative treatment included an elbow brace in a fixed flexion of 10 degrees, which allowed for free rotation of the forearm, for six weeks. Physical therapy with passive movement of the elbow at 0–30 degrees was allowed after two weeks of complete immobilization. After removing the brace, active movement of the elbow was allowed up to the pain threshold, accompanied by further physical therapy. Increasing strength and muscle training was allowed 6–12 weeks after surgery. The tendon was considered fully sustainable six months after the surgery [23]. All patients received this postoperative rehabilitation protocol and underwent physiotherapy in an outpatient setting.

### 2.3. Follow-Up Questionnaire

Demographic information, such as age, height, weight, handedness, treated side, and mechanism of injury, as well as current sporting activities and activities of daily living (ADLs), were evaluated. Sports disciplines were evaluated by analyzing the patients’ pre- and postoperative sportive engagement in 24 different disciplines (plus an open section to name additional activities), frequency of past and currently performed exercises (1, 2, 3, 4, and >4 sessions per week), time of return to sports after the surgery (after six weeks, 4–6 months, 7–12 months, and >12 months, respectively), past and current level of sporting activities (professional, amateur, or none), past and current level of pain (VAS scale, 0–10, 0 meaning the best and 10 meaning the worst result), and current range of motion of the operated elbow (0 degrees, 45 degrees, 90 degrees, 135 degrees, and >135 degrees flexion). Patients who changed sporting activities were asked for the reasons in a free text section. Finally, satisfaction with the surgery in terms of functional and cosmetic outcomes was surveyed (0–10, 0 meaning the best and 10 meaning the worst result).

ADLs were evaluated using several questions concerning difficulties during social, work-related, and everyday activities (no restrictions; some, moderate, or serious problems; inability to perform the task).

### 2.4. Statistical Methods

Statistical analysis was performed using Origin Pro^®^ 9.0 (OriginLab Corp, Northampton, MA, USA) and SPSS^®^ 26.0 (IBM Corp, Armonk, NY, USA). The descriptive study design allows for a lack of power calculation before the study. Descriptive statistics were used for the analysis of the data, and mean values and standard deviations or medians and quartiles were calculated. Independent and paired *t*-tests were used to analyze pre-operative and postoperative data after checking for metric distribution of the data. All data sets were complete.

## 3. Results

### 3.1. Demographics

Forty-four patients were eligible for this study, of whom 22 patients consented to participate and were evaluated using the postal questionnaire (31%). One surgeon performed all surgeries. In all cases but one, the patients were engaged in bodybuilding and/or fitness training. Nine of those individuals were professional athletes.

The mean time of follow-up was 38 months (range, 12–108 months; standard deviation, 2.1), and the mean age at surgery was 44.3 years (median 45 years; range, 28–62 years; SD 9.9). Eleven patients were in each of the two groups, <45 years and ≥45 years of age. All patients were male, and all except one were right-handed. The right elbow was affected in 10 patients, and the left elbow was affected in 12 patients. The indication for surgery in all cases was rupture of the distal triceps tendon, which in most cases (18) occurred during exercise. In the other cases, the causes of rupture included one car accident, one fall unrelated to sporting activities, one accident occurring during manual work, and one accident occurring during gardening. Table 1 shows the demographic values of all patients.

### 3.2. Sports Disciplines

**Figure 2:**
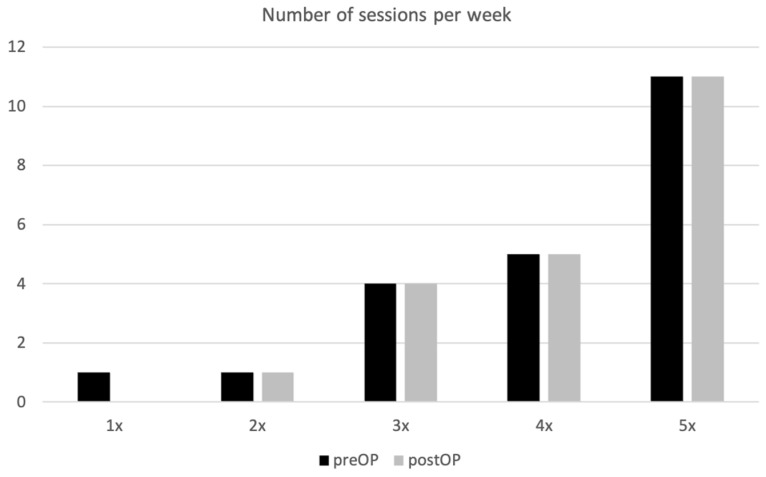
Frequency of exercises. The injury and the subsequent surgery did not have an effect on the number of training sessions per week. Presented as a percentage distribution of the population.

No significant changes were detected comparing the pre-operative (preinjury) and postoperative evaluations of the sports disciplines and the number of sessions per week (Table 2). In general, the subgroup comprising patients younger than 45 years attended more sporting activities and trained slightly more often per week than the older population. However, these differences were not significant. The number of exercise sessions per week did not show significant postoperative deterioration compared with the preinjury state (Figure 2). Overall, ten patients returned to sports after 1.5–3 months, ten patients returned to sports after 4–6 months, and one patient returned to sports 7–12 months after surgery, resulting in an overall return to sports rate of 95% (21/22) after a minimum follow-up of 12 months. In the younger subgroup, three patients returned to sports between 1.5–3 months and seven patients returned to sports after 4–6 months, whereas in the older subgroup, seven patients returned to sports after 6 weeks, and three patients returned to sports after 4–6 months. These differences were not significant (*p* = 0.158).

**Figure 3:**
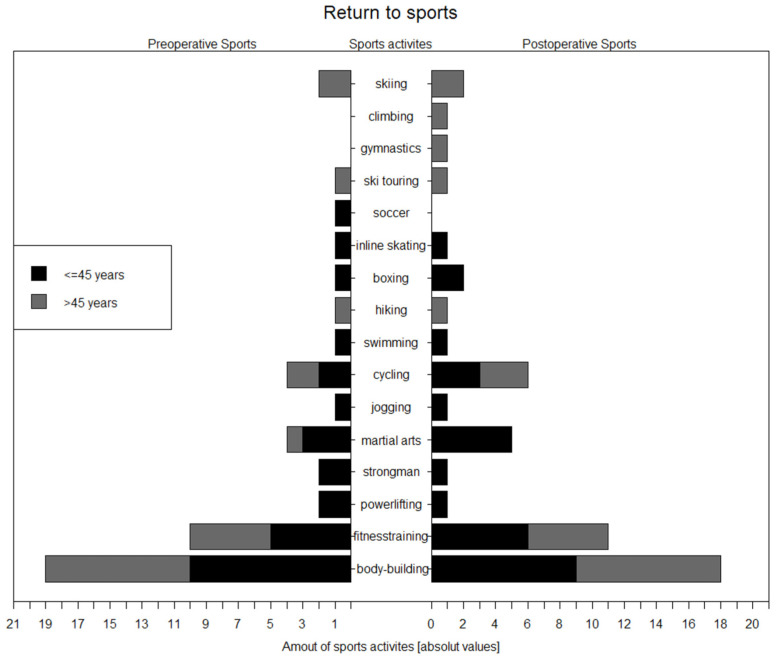
Variety of sporting activities. There was no difference between the two groups in regard to the five most common sporting activities. Presented as absolute values, *n* = 22.

While all patients attended at least one sporting activity, 14 patients had played more than one sport before surgery, with seven in each of the two age groups (younger and older than 45 years). Out of these 14 patients, three changed their sport or reduced the variety, and six decided to change or add a different sporting activity. The reasons for switching disciplines were described as injuries unrelated to triceps repair and changes of interest. One patient who performed a single sporting activity before the injury did not participate in any sports after surgery. All patients who performed competitive bodybuilding before surgery except for two resumed bodybuilding as a sport after surgery. Three patients did not reach the preinjury sporting level, with two of them being over 45 years of age at the time of surgery, leading to a return rate to pre-operative sporting levels of 86%. The five most common sporting activities were bodybuilding, fitness training, cycling, martial arts, and skiing (Figure 3), and there was no difference between the population younger than 45 years and the older population.

### 3.3. Subjective Rating and Well-Being

The elbow pain level before the surgery was low (VAS 0–3) in 29% of the patients, intermediate (VAS 4–6) in 24%, and severe in 47% of the patients (VAS 7–10). After surgery, 90% of the population declared their pain level as low, and 5% each as intermediate and severe. Analyses of pain medication revealed that 36% of patients (8/22) were taking daily pain medication before surgery, whereas the surgery led to a decrease to 0% of daily pain medication at the final follow-up.

The subjective fitness level was rated pre- and postoperatively as a mean level of 5.1 and 2.6 points, respectively (VAS 0–10, *p* = 0.002). There were significant improvements in both subgroups and the overall study population, but no significant age-dependent differences were detected pre-operatively (*p* = 0.952) or postoperatively (*p* = 0.592; Table 3). Only one patient indicated a decrease in his fitness level, and every other patient improved or maintained the pre-operatively measured level.

The ability to attend sports before surgery was rated as 5.2 points, and the surgery led to an increase to 1.3 points (VAS 0–10, *p* < 0.001). While the increase in the overall study population and in the >45 years group was significant, the increase in the younger subgroup was barely significant (Table 3). Every patient experienced an increase from pre- to postoperative or maintained the pre-operative level.

All patients except one had maximum range of motion (elbow extension and flexion, 0-0-150°). Overall, 20 patients subjectively rated their elbow mobility after the surgery as 0–2, and one patient each rated the mobility as 3 and 4.

The subjective satisfaction was excellent, with a satisfaction of 1.5 points (VAS 0–10) for both clinical and cosmetic outcomes. No age-dependent differences were detected (Table 3).

## 4. Discussion

DTTR is a rare but severe injury that leads to reduced function of the upper extremity not only in daily life activities but also in sports. There is a lack of studies that have reported the return-to-sports rate and the sports behavior of competitive strength athletes after surgical repair of the distal tendon of the triceps brachii muscle. This study shows that competitive strength athletes can achieve the same level after surgical repair of DTTR.

In our study, we found an overall return to sports rate of 95% in athletes after surgical treatment. All of our patients were competitive or high-demand bodybuilders with a high eccentric load on their muscles. Compared to Agarwalla et al. [16], this study reports slightly better results. A total of 89.7% of their patients returned to at least one sporting activity by 5.9 (4.4) months following surgery. Our patients returned to sports after a median of 4–6 months postoperatively. However, 29.5% of these patients returned to a lower level of sporting function, resulting in a rate of return to preinjury function of 63%. In our cohort of professional and high-demand bodybuilders, 86% returned to preinjury sporting levels. In the study by Agarwalla et al. [15,16], the patients had various sports habits, and some participants also performed weightlifting, but none were professional bodybuilders, which could explain the difference. When comparing our data to those of professional athletes (NFL players), the latter shows a similar return-to-sports rate of 95% with a smaller sample size (*n* = 10). One could argue that the motivation of professional NFL players is different from that in our cohort (higher financial income, team effort, broader fan attraction), but since comparable results were shown in our cohort, we assume that people who perform sports at a very high level show a greater intrinsic motivation to return to sport [5].

In detail, 94.7% of our patients who performed bodybuilding before surgery continued it afterward. Among our cohort, additional common sports activities besides bodybuilding were fitness training, cycling, martial arts, and skiing. After the surgical intervention, only three patients could not return to the same sports level as they had practiced before the surgery, but they were still participating in sports at the amateur level. However, all other patients returned to the same level of exercise that they had been engaged in pre-operatively. In contrast to our results and despite the comparable results concerning the overall return-to-sports rate, most of the patients examined in the study by Agarwalla et al. [16] were unable to return to the previous level of sport or to their maximal physical performance. On the other hand, a systematic review by Dunn et al. with 262 patients showed a total rate of return to preinjury function of 89% [24]. A more recent systematic review of 591 patients reported a return to preinjury function levels of function in 92% [25]. Furthermore, our patients reported that not only did their subjective fitness level improve postoperatively by 2.6 points on a visual analog scale (pre-operative score of 5.1), but their ability to participate in sports also improved by 1.3 points (pre-operative score of 5.2).

The patients in our study reported a satisfaction of 1.5 points on average on a VAS scale from 0–10. These findings are consistent with a review by Tran et al., who reported that 95% of patients were satisfied with the repair [25].

In our study and in a recent systematic review, it was also shown that males suffer more often from distal triceps tendon rupture [12]. In our study population, we only had males (*n* = 22, 100%), whereas in the study by Alnaji et al. [12], male patients comprised 78.6% of patients (378 of 481), and the mean age of the included participants was 46.1 (8.4) years. This is in line with an older systematic review (mean age: 45.6 years, range: 17–61 years), our population (mean age at surgery of 44.3 years (9.9)) and other studies where DTTR is mostly described for males between 30 and 60 years [1,12,24].

The main risk factors for DTTR are weight training and anabolic steroids [7,24,26]. A meta-analysis of 187 studies performed in different countries reported a prevalence of anabolic use of 6.4% in men and 1.6% in women and a global lifetime prevalence rate of 3.3%. The main reason for the use of anabolic steroids was to increase muscle mass and strength. Although we did not specifically ask regarding the use of androgens, we assume that there was a higher prevalence in our cohort than in a non-bodybuilder cohort due to their well-known effects on muscles [8,27]. In addition, there are further risk factors for DTTR, such as local corticosteroid injection, hyperparathyroidism, renal osteodystrophy, and olecranon bursitis [9,17,18,26,28].

Even though we had no pre-operative clinical scores, our postoperative results in terms of clinical outcomes are quite satisfying compared to other studies. The range of motion showed maximum elbow flexion and extension (S 0-0-150°) in all patients except one (95%, 21/22). Giannicola et al. [18] examined 28 patients (21 men and seven women) with primary repair of DTTR, in which 96.4% similarly achieved a full range of motion in the elbow. Again, a comparison of our cohort to other highly demanding athletes (NFL players) shows that they had similar results in regard to regaining full range of motion [5].

Reviewing the current literature, conservative treatments are recommended only in low-demand patients with partial-thickness tears. In this setting, conservative treatment can lead to successful healing and good clinical results [19,22,26,28,29]. However, for patients with partial tears and a high functional claim, failed conservative treatment, or ruptures with more than 50% tendon disruption, the literature suggests surgical treatment [30,31].

The clinical results after surgical repair of DTTR are quite satisfying not only in our cohort but also in the literature. Nevertheless, there is the risk of postoperative complications and re-rupture. Dunn et al. [24] described a total retear rate of 6%. In their systematic review of 262 patients, they pointed out that renal disease and anabolic steroid use could be risk factors for postoperative re-rupture. A recently performed review of an insurance claims database by Lee et al. showed a complication rate of 5.8% and a one-year revision rate of 2.6% in 8143 patients [2]. After a follow-up on 107 patients, Macknet et al. reported a complication rate of 14% and a reoperation rate of 13.1% in their retrospective analysis [32]. The indications in their population were mostly re-ruptures (9/14). The most described complications in the different literature, however, are not retears but elbow stiffness, neurological problems (ulnar nerve entrapment, posterior interosseous nerve palsy, brachial cutaneous nerve paresthesia, and median nerve paresthesia), heterotopic ossification, anterior arm pain, and infections [9,17,18,20,28]. However, there were no complications or re-ruptures in our patients.

Our hypothesis that surgical repair of DTTR fully restores athletic function in a group of competitive or high-demand bodybuilders was confirmed in this study. In the authors’ opinion, this should lead to a clear recommendation for patients with this injury, in particular for high-performing athletes.

### Limitations

Our study has several limitations. First, the sample size or, more precisely, the number of participants among all eligible patients was small (22/44), which was mostly due to the chosen type of follow-up. Half of the eligible patients could not be contacted or were not willing to participate. This could lead to bias in the results since patients tend to refuse participation if they are not satisfied with the outcome. Second, no pre- or postoperative strength measurements were performed, and there were no patient-reported outcome measures included. Third, the rehabilitation program was not standardized, which was due to a patient-oriented approach because of their strong drive to resume competition. Fourth, the chosen system of investigation was self-reporting by the patients. This can lead to bias because of misunderstanding or error of judgment. The first two points could be addressed via a standardized follow-up protocol for all patients treated, unrelated to research follow-ups. The latter two limitations could best be addressed with a prospective study design with a standardized rehabilitation protocol and evaluation via objective scores, which would be desirable for a better understanding of this rare injury.

## 5. Conclusions

Surgical treatment of DTTR has been shown to be effective in high-demand athletes with a very high rate of return to sports of 95% and a high rate of return to pre-operative sporting level of 86%. The surgery leads to high satisfaction among patients, with a mean of 1.5 on a VAS from 0–10. This surgery can be recommended to competitive or high-demand strength athletes suffering from DTTR with caution due to the study’s imitations.

## Figures and Tables

**Table 1:** Patient Pre-operative Characteristics ^a^.

	<45 Years (*n* = 11)	≥45 Years (*n* = 11)	Overall (*n* = 22)
Gender (m/f)	11/0 (100%/0%)	11/0 (100%/0%)	22/0 (100%/0%)
Age (years)	35.5 (4.4)	53.1 (4.7)	44.3 (9.9)
BMI (kg/m^2^)	30.5 (8.97)	29.4 (2.83)	29.94 (6.71)
Affected side (L/r)	6/5 (55%/45%)	6/5 (55%/45%)	12/10 (55%/45%)
Cause of injury (sports-related/other)	10/1 (91%/9%)	8/3 (73%/27%)	18/4 (82%/18%)

^a^ Data are presented as the mean (standard deviation) or absolute numbers (percentages). m, male; f, female; BMI, body mass index; l, left; r, right.

**Table 2:** Sporting activities and frequency of the study population ^a^.

Subgroup	<45 Years (*n* = 11)	≥45 Years (*n* = 11)	*p*-Value ^b^	Overall (*n* = 22)
Sporting activities				
preOP	2.7 (1.7)	2.0 (0.9)	0.224	2.4 (1.4)
postOP	2.7 (1.9)	2.2 (1.3)	0.427	2.5 (1.6)
*p*-value ^c^	1	0.341		0.540
Frequency (per week)				
preOP	4.2 (1.3)	4.0 (1.0)	0.721	4.1 (1.2)
postOP	4.1 (1.6)	4.0 (1.0)	0.873	4.1 (1.3)
*p*-value ^c^	0.341	1		0.329

^a^ Data are presented as the mean (standard deviation). ^b^ Independent *t*-test. ^c^ Paired *t*-test.

**Table 3:** Pain, level of fitness, ability to train, and satisfaction ^a^.

Subgroup	<45 Years (*n* = 11)	≥45 Years (*n* = 11)	*p*-Value ^b^	Overall (*n* = 22)
VAS pain (0–10)				
preOP	5.5 (3.3)	6.7 (2.6)	0.422	6.0 (3.0)
postOP	1.6 (1.8)	1.6 (1.1)	0.416	1.6 (1.5)
*p*-value ^c^	**0.016**	**<0.001**		**<0.001**
Level of fitness (0–10)				
preOP	5.0 (3.6)	5.3 (3.3)	0.952	5.1 (3.4)
postOP	2.8 (2.2)	2.3 (2.1)	0.592	2.6 (2.1)
*p*-value ^c^	**0.041**	**0.027**		**0.002**
Ability to train (0–10)				
preOP	3.6 (3.4)	7.3 (2.5)	**0.013**	5.2 (3.4)
postOP	1.8 (1.5)	1.1 (0.3)	0.166	1.3 (0.6)
*p*-value ^c^	0.051	**<0.001**		**<0.001**
Satisfaction (0–10)				
Clinical outcome	1.8 (2.4)	1.0 (0.0)	0.386	1.5 (1.9)
Cosmetic outcome	1.7 (1.0)	1.3 (0.5)	0.210	1.5 (0.8)

^a^ Data presented as the mean (standard deviation). ^b^ Independent *t*-test. ^c^ Paired *t*-test. Bold indicates significant differences.

## Data Availability

The data presented in this study are available on request from the corresponding author.

## References

[1] Stucken C., Ciccotti M.G. (2014). Distal Biceps and Triceps Injuries in Athletes. Sports Med. Arthrosc. Rev..

[2] Lee E., Stillson Q.A., Seidel H.D., Bhattacharjee S., Koh J.L., Strelzow J.A., Shi L.L. (2023). Surgical Outcomes, Trends, and Risk Factors of Distal Triceps Repairs. HAND.

[3] Anzel S.H., Covey K.W., Weiner A.D., Lipscomb P.R. (1959). Disruption of Muscles and Tendons; an Analysis of 1014 Cases. Surgery.

[4] Yeh P.C., Dodds S.D., Smart R.L., Mazzocca A.D., Sethi P.M. (2010). Distal Triceps Rupture. Am. Acad. Orthop. Surg..

[5] Mair S.D., Isbell W.M., Gill T.J., Schlegel T.F., Hawkins R.J. (2004). Triceps Tendon Ruptures in Professional Football Players. Am. J. Sports Med..

[6] Stannard J.P., Bucknell A.L. (1993). Rupture of the Triceps Tendon Associated with Steroid Injections. Am. J. Sports Med..

[7] Visuri T., Lindholm H. (1994). Bilateral Distal Biceps Tendon Avulsions with Use of Anabolic Steroids. Med. Sci. Sports Exerc..

[8] García-Arnés J.A., García-Casares N. (2022). Doping and Sports Endocrinology: Anabolic-Androgenic Steroids. Rev. Clín. Esp. (Engl. Ed.).

[9] van Riet R.P., Morrey B.F., Ho E., O’Driscoll S.W. (2003). Surgical Treatment of Distal Triceps Ruptures. J. Bone Jt. Surg. Am..

[10] Horneff J.G., Aleem A., Nicholson T., Lervick G., Murthi A., Sethi P., Getz C., Lazarus M.D., Ramsey M.L., Abboud J.A. (2017). Functional Outcomes of Distal Triceps Tendon Repair Comparing Transosseous Bone Tunnels with Suture Anchor Constructs. J. Shoulder Elbow Surg..

[11] Mirzayan R., Acevedo D.C., Sodl J.F., Yian E.H., Navarro R.A., Anakwenze O., Singh A. (2018). Operative Management of Acute Triceps Tendon Ruptures: Review of 184 Cases. Am. J. Sports Med..

[12] Alnaji O., Erdogan S., Shanmugaraj A., AlQahtani S., Prada C., Leroux T., Khan M. (2022). The Surgical Management of Distal Triceps Tendon Ruptures: A Systematic Review. J. Shoulder Elbow Surg..

[13] Dorweiler M.A., Van Dyke R.O., Siska R.C., Boin M.A., DiPaola M.J. (2017). A Comparative Biomechanical Analysis of 2 Double-Row, Distal Triceps Tendon Repairs. Orthop. J. Sports Med..

[14] Yeh P.C., Stephens K.T., Solovyova O., Obopilwe E., Smart L.R., Mazzocca A.D., Sethi P.M. (2010). The Distal Triceps Tendon Footprint and a Biomechanical Analysis of 3 Repair Techniques. Am. J. Sports Med..

[15] Agarwalla A., Gowd A.K., Jan K., Liu J.N., Garcia G.H., Naami E., Wysocki R.W., Fernandez J.J., Cohen M.S., Verma N.N. (2021). Return to Work Following Distal Triceps Repair. J. Shoulder Elbow Surg..

[16] Agarwalla A., Gowd A.K., Liu J.N., Garcia G.H., Jan K., Naami E., Wysocki R.W., Fernandez J.J., Cohen M.S., Verma N.N. (2022). Return to Sport Following Distal Triceps Repair. J. Hand Surg. Am..

[17] Blackmore S.M., Jander R.M., Culp R.W. (2006). Management of Distal Biceps and Triceps Ruptures. J. Hand Ther..

[18] Giannicola G., Bullitta G., Rotini R., Murena L., Blonna D., Iapicca M., Restuccia G., Merolla G., Fontana M., Greco A. (2018). Results of Primary Repair of Distal Triceps Tendon Ruptures in a General Population: A Multicentre Study. Bone Jt. J..

[19] Demirhan M., Ersen A. (2016). Distal Triceps Ruptures. EFORT Open Rev..

[20] Gaviria M., Ren B., Brown S.M., McCluskey L.C., Savoie F.H., Mulcahey M.K. (2020). Triceps Tendon Ruptures. JBJS Rev..

[21] Khiami F., Tavassoli S., De Ridder Baeur L., Catonné Y., Sariali E. (2012). Distal Partial Ruptures of Triceps Brachii Tendon in an Athlete. Orthop. Traumatol. Surg. Res..

[22] Bain G.I., Durrant A.W. (2010). Sports-Related Injuries of the Biceps and Triceps. Clin. Sports Med..

[23] Ritsch M., Regauer M., Schoch C. (2022). Surgical Treatment of Distal Triceps Tendon Ruptures. Oper. Orthop. Traumatol..

[24] Dunn J.C., Kusnezov N., Fares A., Rubin S., Orr J., Friedman D., Kilcoyne K. (2017). Triceps Tendon Ruptures: A Systematic Review. Hand.

[25] Tran D.V., Yetter T.R., Somerson J.S. (2022). Surgical Repair of Distal Triceps Rupture: A Systematic Review of Outcomes and Complications. JSES Rev. Rep. Tech..

[26] Walker C.M., Noonan T.J. (2020). Distal Triceps Tendon Injuries. Clin. Sports Med..

[27] Sagoe D., Molde H., Andreassen C.S., Torsheim T., Pallesen S. (2014). The Global Epidemiology of Anabolic-Androgenic Steroid Use: A Meta-Analysis and Meta-Regression Analysis. Ann. Epidemiol..

[28] Kose O., Kilicaslan O.F., Guler F., Acar B., Yuksel H.Y. (2015). Functional Outcomes and Complications after Surgical Repair of Triceps Tendon Rupture. Eur. J. Orthop. Surg. Traumatol..

[29] Harris P.C., Atkinson D., Moorehead J.D. (2004). Bilateral Partial Rupture of Triceps Tendon. Am. J. Sports Med..

[30] Balazs G.C., Brelin A.M., Dworak T.C., Brooks D.I., Mauntel T.C., Tintle S.M., Dickens J.F. (2016). Outcomes and Complications of Triceps Tendon Repair Following Acute Rupture in American Military Personnel. Injury.

[31] Freislederer F., Papillo D., Glanzmann M., Scheibel M. (2020). Distale Bizepssehnen- und Trizepssehnenrupturen. Z. Orthop. Unfall.

[32] Macknet D.M., Ford S.E., Mak R.A., Loeffler B.J., Connor P.M., Gaston R.G. (2022). Complications after Traumatic Distal Triceps Tears: An Analysis of 107 Cases. JSES Rev. Rep. Tech..

